# Preparation of Graphene Oxide-Based Hydrogels as Efficient Dye Adsorbents for Wastewater Treatment

**DOI:** 10.1186/s11671-015-0931-2

**Published:** 2015-06-27

**Authors:** Haiying Guo, Tifeng Jiao, Qingrui Zhang, Wenfeng Guo, Qiuming Peng, Xuehai Yan

**Affiliations:** State Key Laboratory of Metastable Materials Science and Technology, Yanshan University, Qinhuangdao, 066004 People’s Republic of China; Hebei Key Laboratory of Applied Chemistry, School of Environmental and Chemical Engineering, Yanshan University, Qinhuangdao, 066004 People’s Republic of China; National Key Laboratory of Biochemical Engineering, Institute of Process Engineering, Chinese Academy of Sciences, Beijing, 100190 People’s Republic of China

**Keywords:** Graphene oxide, Hydrogel, Nanostructures, Polymer, Dye removal

## Abstract

Graphene oxide (GO) sheets exhibit superior adsorption capacity for removing organic dye pollutants from an aqueous environment. In this paper, the facile preparation of GO/polyethylenimine (PEI) hydrogels as efficient dye adsorbents has been reported. The GO/PEI hydrogels were achieved through both hydrogen bonding and electrostatic interactions between amine-rich PEI and GO sheets. For both methylene blue (MB) and rhodamine B (RhB), the as-prepared hydrogels exhibit removal rates within about 4 h in accordance with the pseudo-second-order model. The dye adsorption capacity of the hydrogel is mainly attributed to the GO sheets, whereas the PEI was incorporated to facilitate the gelation process of GO sheets. More importantly, the dye-adsorbed hydrogels can be conveniently separated from an aqueous environment, suggesting potential large-scale applications of the GO-based hydrogels for organic dye removal and wastewater treatment.

## Background

Nowadays, harmful chemical compounds have become the main cause of water pollution. Water pollution exerts negative effects not only on species living in the water but also on the broader biological community. For instance, organic dyes are often discharged with wastewater into the local environment without adequate treatment. Rapid and convenient removal of organic dyes from wastewater has been a challenging issue faced by scientists [1–6]. For example, Kim’s groups achieved excellent systematic works in the relative fields of water remediation by various nanocomposites [1, 2]. In particular, large-scale application requires the potential dye adsorbents to exhibit a high dye removal rate within a relatively short period of time and to be environmentally friendly. For the latter, the adsorbents must be able to be properly separated from an aqueous environment after adsorbing waste dyes. In the past years, graphene oxide (GO) sheets have attracted broad attention as potential dye adsorbents because of their unique conjugated, two-dimensional (2D) structure, which exhibits superior adsorption capacity for various dye molecules through π-π stacking interactions [7–12]. In addition, the negative charges in the GO sheets due to various oxygen-rich functional groups (i.e., carboxy, carbonyl, hydroxyl groups) allow additional strong electrostatic interactions with cationic dye molecules [13–18]. However, GO sheets exhibit a high dispersibility in water, which prevents the efficient separation of dye-adsorbed GO sheets from an aqueous environment. Therefore, various GO-based adsorbent materials have been developed to facilitate the separation of dye-adsorbed GO sheets from aqueous solutions [19–21]. For example, Akhavan et al. successfully reported the preparation and magnetic separation application of superparamagnetic ZnFe_2_O_4_/reduced graphene oxide (rGO) composites by hydrothermal reaction method [22]. In addition, their group has also investigated some bacteria bioactivity and interaction with the environment by aggregated graphene nanosheets as an encapsulating material and effective photothermal agent [23]. In addition, depositing magnetic Fe_3_O_4_ nanoparticles on GO sheets can allow facile separation of dye-adsorbed composites by applying an external magnetic field [19]. GO-based porous materials have also been used to adsorb organic waste dyes [21]. Alternatively, GO-based hydrogels provide an effective solution for the easy separation of dye-adsorbed materials from water [24].

In this work, the facile preparation of GO/polyethylenimine (PEI) hydrogels as efficient dye adsorbents for wastewater treatment was reported. The GO/PEI hydrogels were obtained through both hydrogen bonding and electrostatic interactions between amine-rich PEI and GO sheets. PEI was incorporated to facilitate the gelation process of GO sheets, and the dye adsorption capacity of the hydrogel is mainly attributed to the GO sheets. For both methylene blue (MB) and rhodamine B (RhB), the as-prepared hydrogels exhibit removal rates within 4 h in accordance with the pseudo-second-order model. More importantly, the dye-adsorbed hydrogels can be conveniently separated from an aqueous environment, suggesting potential large-scale applications of the GO-based hydrogels for organic dye removal and wastewater treatment.

## Methods

**Fig. 1:**
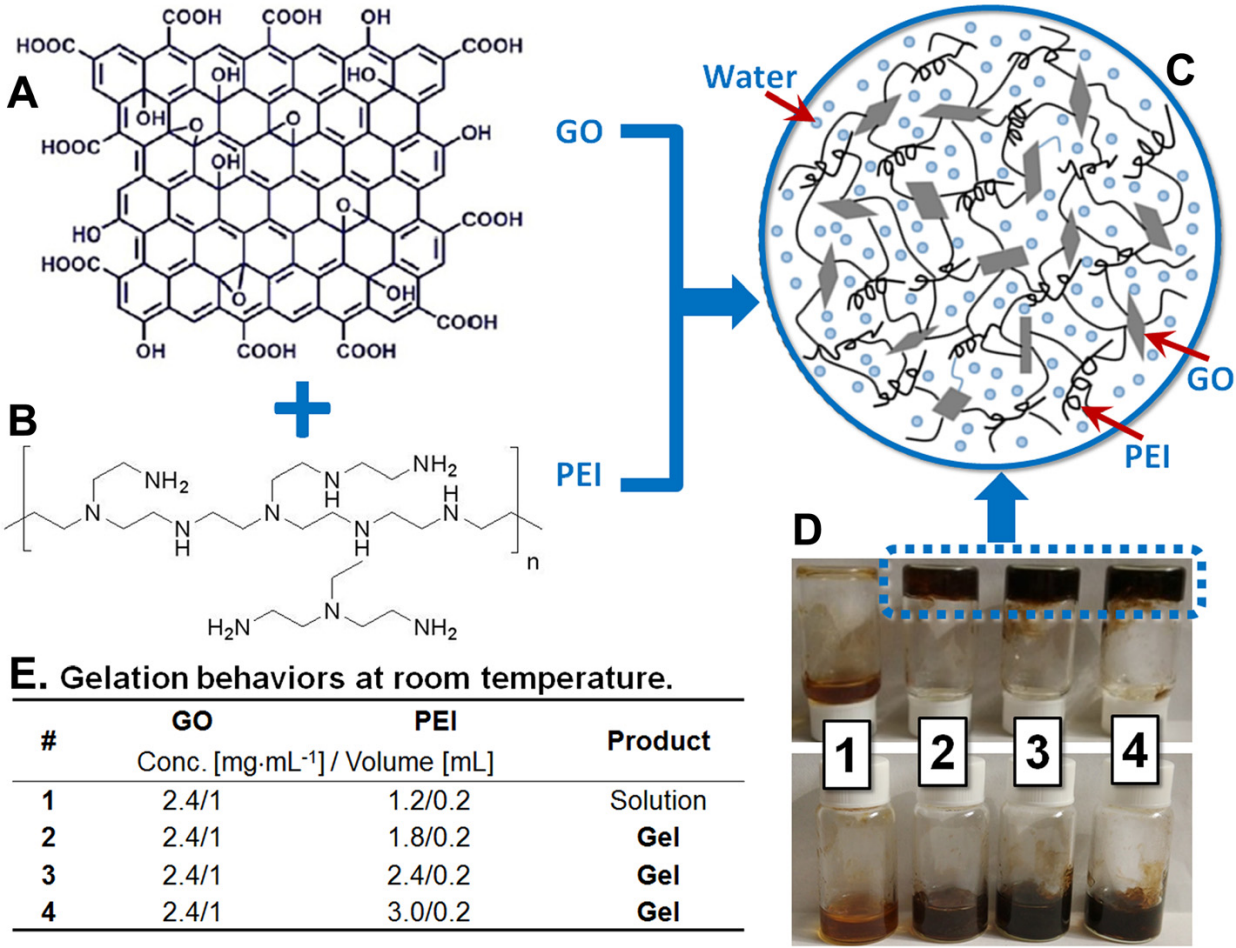
Schematic depiction of the formation of GO/PEI gels. **a** GO and **b** amine-rich PEI were combined to give **c** GO/PEI hydrogels. **d**, **e** Gelation pictures

The starting materials, PEI (Mw = 600 g·mol^−1^, Aladdin Reagent, Shanghai, China), RhB (Tianjin Kaitong Chemical Reagent Co., Ltd, Tianjin, China), and MB (Tianjin Kaitong Chemical Reagent Co., Ltd., Tianjin, China), were used as received. GO sheets were prepared according to the method described by Hummer [25] with some modification [26]. Deionized (DI) water was used in all cases. PEI was first dissolved in DI water to make aqueous stock solutions with different concentrations (1.2, 1.8, 2.4, and 3.0 mg·mL^−1^). GO powder (24 mg) was dispersed in 10 mL of DI water to give a stock solution (2.4 mg·mL^−1^). GO/PEI hydrogels were prepared by combining GO and PEI stock solutions with sonication for a few seconds or without sonication for a few minutes to form gels. The detailed formulations are listed in Fig. 1e. The prepared samples of the solution and hydrogels were designated as #1, #2, #3, and #4.

The presently used different xerogels were obtained at −50 °C via a lyophilizer (FD-1C-50, Beijing Boyikang Experimental Instrument Co., Ltd., China) to completely remove water over 2–3 days. The morphology of GO and lyophilized GO/PEI hydrogels was characterized by using both field-emission scanning electron microscopy (FE-SEM, S-4800II, Hitachi, Japan) with an accelerating voltage of 5–15 kV and transmission electron microscopy (TEM, HT7700, Hitachi High-Technologies Corporation) with commercial 300-mesh copper grids. Before SEM investigations, the prepared samples were coated with copper foil fixed by a conductive adhesive tape and covered with gold nanoparticles to make them more conductive. X-ray diffraction study was carried out by using an X-ray diffractometer (SmartLab, Rigaku, Japan) equipped with a conventional Cu Kα X-ray radiation (*λ* = 1.54 Å) source and a Bragg diffraction setup. Transmission Fourier transform infrared (FT-IR) spectra were obtained using a Nicolet iS10 FT-IR spectrophotometer from Thermo Fisher Scientific Inc. (Waltham, MA, USA) with an average of 16 scans and at a resolution of 4 cm^−1^ by the conventional KBr disk tablet method. Thermogravimetry-differential scanning calorimetry (TG-DSC) analyses of the samples were conducted in air condition by using a Netzsch STA 409 PC Luxx simultaneous thermal analyzer (Netzsch Instruments Manufacturing Co., Ltd., Germany). Raman spectroscopy was performed using a Horiba Jobin Yvon Xplora PLUS confocal Raman microscope equipped with a motorized sample stage. The wavelength of the excitation laser was 532 nm, and the power of the laser was kept below 1 mW without noticeable sample heating. The intensity of a Raman peak was extracted from the maximum value after baseline subtraction over the corresponding spectral range. X-ray photoelectron spectroscopy (XPS) was performed on Thermo Scientific ESCALAB 250Xi using 200-W monochromated Al Kα radiation. The 500-μm X-ray spot was used for XPS analysis. The base pressure in the analysis chamber was about 3 × 10^−10^ mbar. Typically, the hydrocarbon C(1s) line at 284.8 eV from adventitious carbon is used for energy referencing. Both survey scan and individual high-resolution scan peaks were recorded.

The adsorption experiments were designed and modified according to the previous reports [27, 28]. In adsorption experiments, about 1 mL of GO/PEI hydrogel (without lyophilizing) was added to 100 mL of either MB (10 mg·L^−1^) or RhB (4 mg·L^−1^) solutions. The dye solutions containing gel adsorbents were stirred slowly and continuously at room temperature in a dark condition. The gel samples were then separated by centrifugation at different time intervals, and the supernatant liquid was collected for subsequent analysis using an UV-vis spectrometer (752, Sunny Hengping, Shanghai, China). The absorbance at 662 nm (MB) and 554 nm (RhB) was used to determine the concentration of residual dyes in the supernatant liquid.

## Results and discussion

**Fig. 2:**
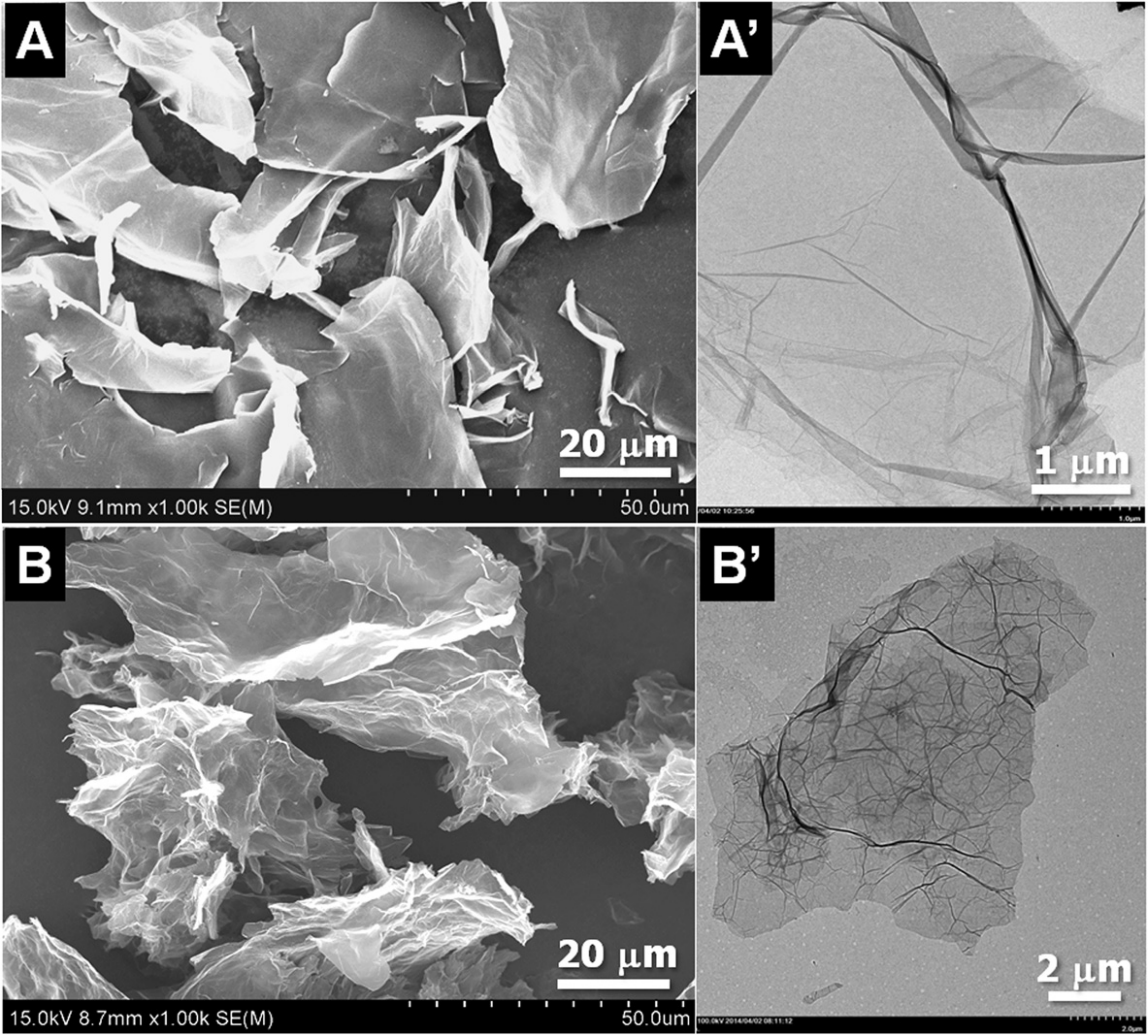
Morphology of lyophilized GO sheets (**a**, **a’**) and GO/PEI gel (**b**, **b’**). SEM images (**a**, **b**) and TEM images (**a’**, **b’**)

Figure 1 depicts the complete preparation process of GO/PEI hydrogels by combining the GO suspension and the PEI aqueous solution using the formulation listed in Fig. 1e. The prepared samples were designated as #1, #2, #3, and #4. GO sheets are rich in hydrophilic functional groups (e.g., carboxyl, hydroxyl, and epoxides) (Fig. 1a). Functional groups like –OH and –COOH can form hydrogen bonds with amines or amine-containing molecules under appropriate conditions [16, 29, 30]. Therefore, amine-rich PEI (Fig. 1b) was chosen to facilitate the gelation of GO sheets in an aqueous solution. In addition, PEI also exhibits good adsorption and adhesion properties [31]. It was found that the dye adsorption capacity of the above porous materials increases with the amount of PEI content, presumably due to the strong electrostatic attractions between the amine-rich PEI chains and the dye molecules. Therefore, the adsorption capacity of the abovementioned porous materials is essentially attributed to PEI instead to GO. On the other hand, it is well known that in most composite materials, as a kind of functional molecule with multi-amine groups, PEI was used widely as a strong chelating agent and organic intermediate. As for the present as-formed GO-based composite gels, after combination with GO, the formed composites showed very stable self-assembly behaviors by strong hydrogen bonding and electronic interactions, which showed a rare possibility to release PEI to cause secondary waste as dye adsorbents for wastewater treatment. In this study, PEI was only used with a concentration slightly above the critical gelation concentration (i.e., the minimum concentration of the gelator needed for gel formation). The formulation of the GO/PEI gels is shown in Fig. 1e, and photos of the GO/PEI gels are shown in Fig. 1d. The samples possessing good gelation properties are designated as #2, #3, and #4 and were used in the dye adsorption experiments as discussed later in this paper. In addition, the morphology of GO and lyophilized GO/PEI hydrogels was characterized using both FE-SEM and TEM. Figure 2a, a’ shows a typical 2D flake-like morphology of GO sheets. Figure 2b, b’ reveals the porous microstructures of lyophilized GO/PEI gels, suggesting that the GO sheets were cross-linked in the porous PEI networks.

**Fig. 3:**
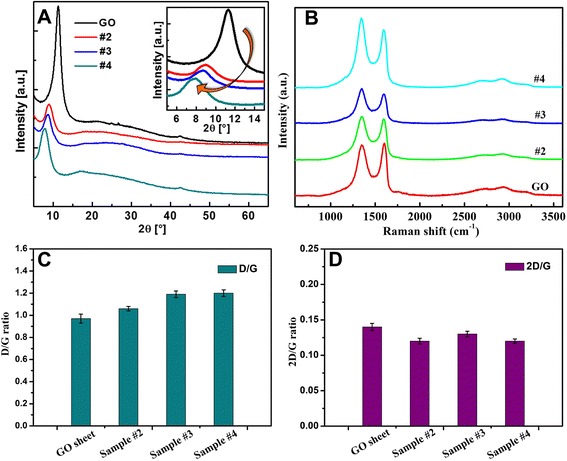
X-ray diffraction patterns (**a**) and Raman spectra (**b**) of GO and lyophilized GO/PEI hydrogels (#2, #3, and #4). **c**, **d** D/G and 2D/G ratios of the Raman spectra shown in **b**, respectively

In addition, the strong affiliation between PEI and GO was also evidenced by X-ray diffraction studies. Figure 3a shows the diffraction patterns for the GO and the lyophilized GO/PEI hydrogels. The 2*θ* values were observed at 11.3° (GO), 8.9° (gel #2), 8.6° (gel #3), and 7.8° (gel #4), corresponding to the *d*-spacing values of 0.77, 0.98, 1.02, and 1.13 nm, respectively. The X-ray patterns of GO display the presence of a strong peak at 11.3° corresponding to the (001) reflection peak with a layer distance of 0.77 nm [32]. Thus, the regular stacking of GO sheets was significantly altered by PEI chains attached on the surface of the GO sheets, even though the structural features of GO remained largely unchanged. Moreover, Raman spectroscopy provides a useful tool to characterize the carbon-based materials [33], as shown in Fig. 3b. Three characteristic bands of graphene sheets in Raman spectra appeared, including the G band (1601 cm^−1^) originated from the first-order scattering of the E_2_g phonons of the sp^2^-hybridized carbon atoms, the D band (1351 cm^−1^) caused by a breathing mode of κ-point phonons of A_1_g symmetry of the defects involved in the sp^3^-hybridized carbon bonds such as hydroxyl and/or epoxide bonds [34], and the 2D band (2692 cm^−1^) which is much sensitive to stacking of graphene sheets [35]. It is established that the G and 2D bands of single-layer graphene sheets are usually located at 1585 and 2679 cm^−1^, while for multi-layer graphene sheets (including 2–6 layers), the positions of the G and 2D bands shift into lower and higher wavenumbers, respectively [36, 37]. Furthermore, the 2D/G ratios of single-, double-, triple-, and multi-layer (>4) graphene sheets are typically >1.6, 0.8, 0.30, and 0.07, respectively [38]. For example, Akhavan achieved excellent research work and reported successfully the 2D/G ratios of the single and bilayer GO sheets in the range of 1.53–1.68 and 0.82–0.89, respectively [39]. In our present work, the 2D/G ratios of the GO sheets and three different composite gels showed the values in the range of 0.12–0.14 (seen in Fig. 3d), suggesting the multi-layer nature of the presently prepared graphene sheets. In addition, due to the origination of the G and D bands, the G/D peak intensity ratio is known as a measure of the sp^2^ domain size of graphene sheets containing sp^3^ and sp^2^ bonds. In our present work, it was found that by forming the composite gels, the D/G ratios (shown in Fig. 3c) shifted from 0.97 to the values of 1.06, 1.19, and 1.20 with increment of PEI concentration, respectively. This change can be attributed to the successful cross-linking of GO in the hydrogel networks and the absence of the C–N bonds formed on the surface of the GO sheets.

**Fig. 4:**
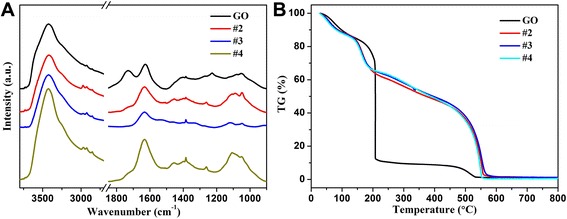
IR spectra (**a**) and TG curves (**b**) of GO and lyophilized GO/PEI hydrogels (#2, #3, and #4)

The FT-IR spectra of GO and GO/PEI hydrogels are demonstrated in Fig. 4a. In the spectrum of GO, the peak at 3432 cm^−1^ could be assigned to the –OH vibration stretching. It also showed bands due to carboxyl C=O (1724 cm^−1^), epoxy C–O (1226 cm^−1^), and alkoxy C–O (1050 cm^−1^) groups situated at the edges of the GO nanosheets [16, 29, 30]. In the spectra of GO/PEI gels, an obvious peak could be observed at 1645 cm^−1^, corresponding to the –NH stretching of polyethylenimine. In addition, the appearance of the bands at 2920 and 2846 cm^−1^ was also observed, which could be assigned to the methyl stretching in PEI molecules. The obtained FT-IR results clearly indicated that GO-based composite hydrogels have been successfully prepared. In addition, Fig. 4b illustrates the thermograms of GO and GO-based composite hydrogels. The quality of GO declines uniformly from 30 to 150 °C, and the loss of the quality is about 15 %, which is mainly due to the moisture evaporation of the sample. With increment of temperature, the quality of GO declines sharply, especially at 207 °C. This can be due to the pyrolysis of the unstable oxygen-containing functional groups in GO. When the temperature is higher than 450 °C, GO tends to produce further losses. Moreover, according to TG results, GO/PEI hydrogels showed a higher thermal stability compared with the GO sheet, which could be attributed to the higher cross-linking within the network structures in gels. Because of the effect of PEI, it can be found that addition of a low content of PEI can greatly increase the thermal stability of hydrogels, suggesting that a strong interaction exists between GO sheets and PEI even in such a water-abundant hydrogel. It was reported that some GO composites showed different weight retention values at high temperature, probably due to the structural changes from the existence of the carbon net-compound assembly structure in the composites [40–42]. In the present case, the as-formed composite hydrogels enhance the thermal stability of materials to a certain extent.

**Fig. 5:**
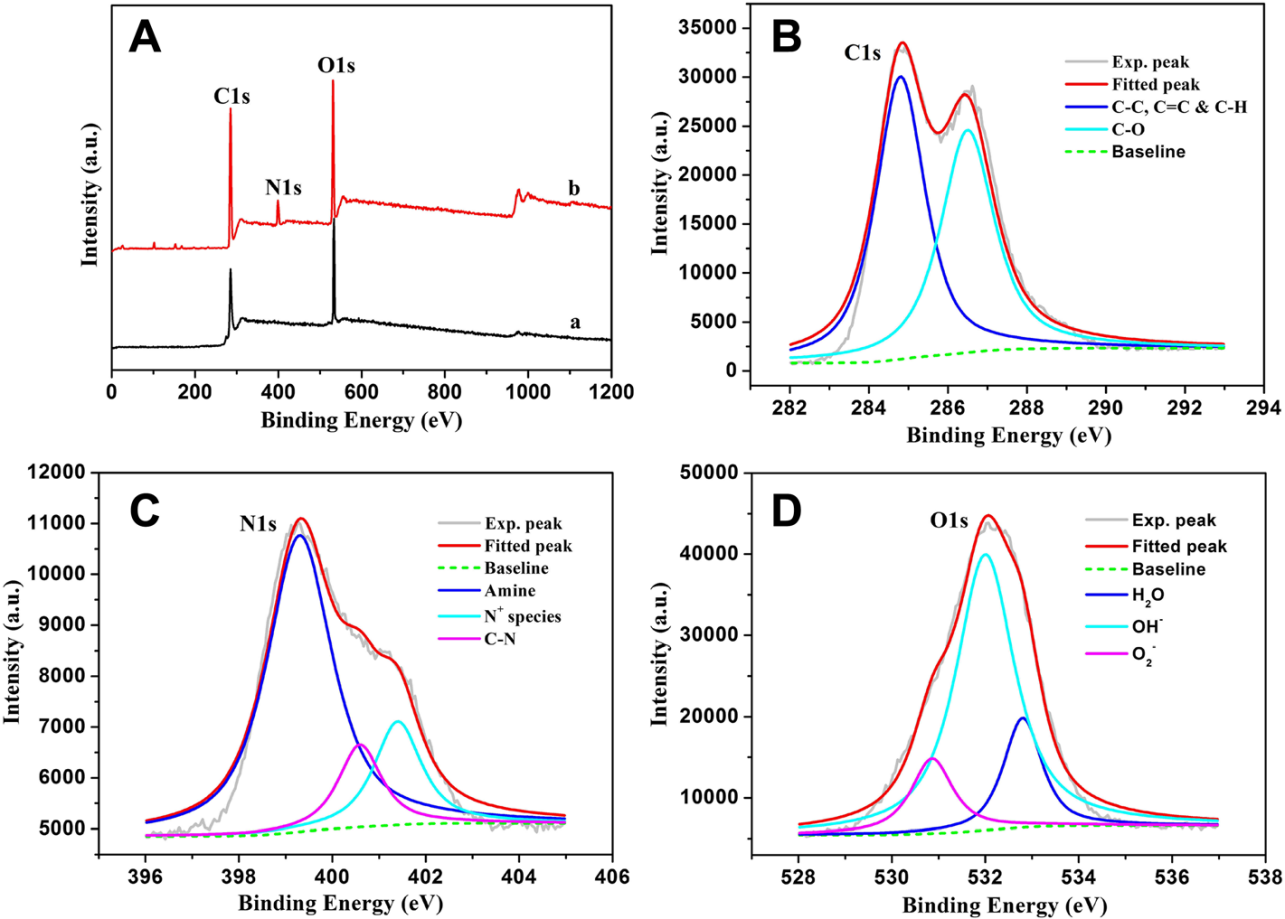
Survey XPS spectra (**a**) of samples: *a*, GO sheet; *b*, GO/PEI hydrogel (#2). Deconvolution of XPS peaks of the GO/PEI hydrogel (#2) nanocomposite: **b** C(1s), **c** N(1s), and **d** O(1s)

In order to further investigate the obtained GO/PEI hydrogel, the survey XPS spectra of the lyophilized GO/PEI hydrogel (#2) in Fig. 5a showed the characteristic peaks, such as C(1s), N(1s), and O(1s). In addition, we obtained the relative elemental composition and calculated the O/C ratios of all lyophilized samples (GO sheet, 37.26 %; GO/PEI gel, 36.08 %), respectively, which suggested the decrement of the oxygen element from GO to the nanocomposite. Moreover, the deconvolution of C(1s), N(1s), and O(1s) of XPS peaks for the GO/PEI hydrogel (#2) nanocomposite was demonstrated. Figure 5b shows XPS peak deconvolution of C(1s) core levels of the gel (#2) nanocomposite. The peak centered at 284.9 eV was attributed to the C–C, C=C, and C–H bonds. The other deconvoluted peak located at the binding energies of 286.7 eV was assigned to the C–OH oxygen-containing bonds [43]. The high-resolution N(1s) spectrum in Fig. 5c reveals the presence of amine (399.4 eV), C–N bond (400.6 eV), and N^+^ species (401.4 eV), suggesting the presence of PEI polymers in the composite, either in their original amine forms or in grafted forms through the covalent bonding and weak interaction force to the GO sheets [44]. In addition, the O(1s) photoelectron peak of the gel (#2) nanocomposite is shown in Fig. 5d. This peak can be deconvoluted into three Gaussian components with identical FWHM after a Shirley background subtraction. The second component at 532.0 eV can be corresponded to the oxygen of the surface OH^−^ bound in nanocomposite [45]. The third deconvoluted O(1s) peak at 532.9 eV was attributed to the oxygen of water molecules existing in the nanostructure or adsorbed on the GO surface. This means that the surface of the gel (#2) nanocomposite was still porous, which can be an advantage for the surface adsorption process.

**Fig. 6:**
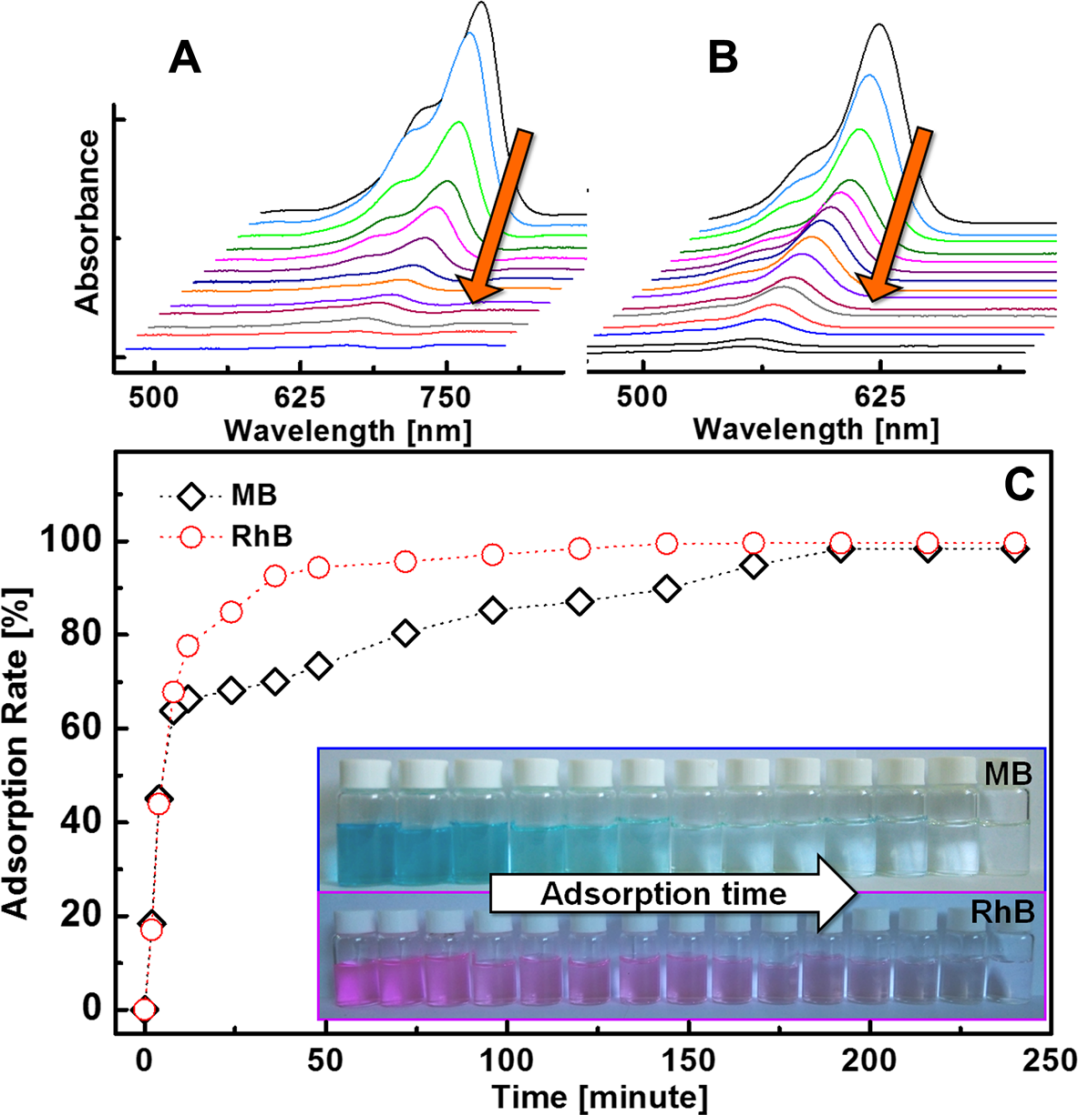
The absorption spectra for MB (**a**) and RhB (**b**) acquired for the supernatant liquids collected at different time intervals during dye adsorption experiments. The calculated dye removal rates versus time plots for both MB and RhB (**c**)

**Fig. 7:**
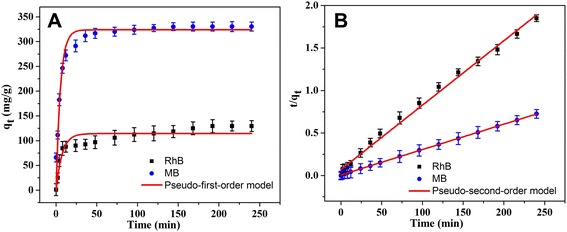
Adsorption kinetics curves of as-prepared GO/PEI hydrogel (#2) on MB and RhB at 298 K. **a** Pseudo-first-order kinetics. **b** Pseudo-second-order kinetics

The dye adsorption capacity was evaluated by placing the as-prepared GO/PEI hydrogels in MB and RhB aqueous solutions. It should be noted that the adsorption process of freeze-dried samples seemed typical and easy to investigate. In the present work, the in situ adsorption behaviors of hydrogels were chosen, which could demonstrate the real adsorption process of GO-based gels for different dyes in wastewater. In addition, graphene oxide has the probability to act as a visible light photocatalyst for degradation of dyes in the adsorption process. So the present adsorption experiments were measured and repeated in a dark condition. The absorbance at 662 nm (Fig. 6a for MB) and 554 nm (Fig. 6b for RhB) was used to determine the concentration of residual dyes for samples collected at different time intervals. The dye removal rates were calculated according to the equation of *K* = (*A*_0_ − *A*_T_)/*A*_0_ × 100 %, where *K* is the dye removal rate, *A*_0_ is the absorbance of the dye stock solution, and *A*_T_ is the absorbance of the supernatant liquid collected at different time intervals. Figure 6c shows the calculated dye removal rate versus time plots for both MB and RhB. The dye removal rates can reach nearly 100 % for both MB and RhB within approximately 4 h, suggesting the as-prepared GO/PEI hydrogels as efficient dye adsorbents. In addition, the thermodynamic behaviors of other reduced graphene oxide-based hydrogels for dye adsorption from aqueous solutions were reported and investigated in detail [46]. Now our primary adsorption kinetic experiments of the as-prepared GO/PEI hydrogel (#2) on MB and RhB were performed, and the results are shown in Fig. 7. The hydrogel exhibits a continuous adsorption process, with equilibrium times of approximately 200 min for MB and RhB, respectively. A 200-min equilibrium time is acceptable for efficient photocatalytic applications. Such kinetic behavior can also be associated with the unique nanocomposite structure, i.e., the large three-dimensional network-like nanostructure cross-linked with polyethylenimine by electrostatic attractions and hydrogen bonding, and highly dispersed GO nanosheets as the adsorption sites. In addition, classical kinetic models were employed to describe the above degradation data as follows:

The pseudo-first-order model:

1 $$ \log \left({q}_e-{q}_t\right)= \log {q}_e-\frac{k}{2.303}t $$

The pseudo-second-order model:

2 $$ \frac{t}{q_{\mathrm{t}}}=\frac{1}{k{q_{\mathrm{e}}}^2}+\frac{t}{q_{\mathrm{e}}} $$

where *q*_e_ and *q*_t_ represent the amount of dye adsorbed (mg/g) at equilibrium and time *t*, respectively, and the *k*_1_ or *k*_2_ values are the kinetic rate constants. The kinetic data (Table 1) can be accurately described by the pseudo-second-order model with a high correlation coefficient (*R*^2^ > 0.994). In addition, it should be noted that only a slightly high concentration of PEI above the gelation condition was chosen and used. Thus, present types of composite materials could not be reused many times with poor recycling ability. The design of stabilized GO-based hydrogel materials and the relative applications are still a challenging problem in the near future.

**Table 1:** Kinetic parameters of GO/PEI hydrogel (#2) for MB and RhB adsorptions at 298 K (experimental data from Fig. 7)

GO/PEI hydrogel (#2)	Pseudo-first-order model			Pseudo-second-order model		
	*q*_e_ (mg/g)	*R* ^2^	*K*_1_ (min^−1^)	*q*_e_ (mg/g)	*R* ^2^	*K*_2_ (g/min·h)
MB	323.9481	0.94487	0.18890	334.448	0.99987	0.001140
RhB	114.4099	0.88575	0.13647	131.926	0.99458	0.000808

Considering the obtained experimental results described above, some important points should be proposed and discussed. Firstly, in our recent works about some organogel systems based on organic compounds [47–51], functionalized imide derivatives, with the different substituent groups (such as cholesteryl, azobenzene, or luminol), molecular skeletons, or spacers, can have a profound effect on the gelation abilities and the as-formed nanostructures. In another organogel system based on cationic amphiphile-GO nanocomposites, the headgroups in amphiphiles play a crucial role in the gelation behaviors in various organic solvents [52]. For the present GO/PEI hydrogels, the self-assembly and regular stacking of GO sheets were significantly altered by formulation of PEI attached on the surface of GO sheets. In addition, it should be noted that the present GO/PEI hydrogels are more environmentally friendly than organogels from different organic solvents. Now the drug release behaviors and preparation of nanoparticle-containing hybrid hydrogels generated by the present supramolecular gels are under investigation to display the relationship between the as-formed nanostructures and their applications.

## Conclusions

In summary, the facile preparation and dye adsorption capacity of GO/PEI hydrogels have been investigated. PEI was chosen for its abundant amine groups that can form hydrogen bonds with GO. Both the SEM and XRD studies clearly show that the GO sheets were successfully cross-linked in the PEI network. Meanwhile, the Raman spectra suggest that the structural features of GO sheets remain largely unchanged pre- and post-gelation. The as-prepared GO/PEI hydrogels exhibited good removal rates for both MB and RhB in accordance with the pseudo-second-order model. The current research work provides further insight into the applications of GO-based polymer-containing hydrogels as dye adsorbents for wastewater treatment.
